# Commercial toilets emit energetic and rapidly spreading aerosol plumes

**DOI:** 10.1038/s41598-022-24686-5

**Published:** 2022-12-08

**Authors:** John P. Crimaldi, Aaron C. True, Karl G. Linden, Mark T. Hernandez, Lars T. Larson, Anna K. Pauls

**Affiliations:** Department of Civil, Environmental, and Architectural Engineering, University of Colorado, Boulder, CO 80309 USA

**Keywords:** Engineering, Applied physics

## Abstract

Aerosols can transmit infectious diseases including SARS-CoV-2, influenza and norovirus. Flushed toilets emit aerosols that spread pathogens contained in feces, but little is known about the spatiotemporal evolution of these plumes or the velocity fields that transport them. Using laser light to illuminate ejected aerosols we quantify the kinematics of plumes emanating from a commercial flushometer-type toilet, and use the motion of aerosol particles to compute velocity fields of the associated flow. The toilet flush produces a strong chaotic jet with velocities exceeding 2 m/s; this jet transports aerosols to heights reaching 1.5 m within 8 seconds of initiating a flush. Quantifying toilet plumes and associated flow velocities provides a foundation for future design strategies to mitigate plume formation or to disinfect pathogens within it.

## Introduction

Flushing a toilet generates an energetic turbulent flow that releases droplets and aerosols into the air^1–4^, reaching heights in excess of 1.5 m^5^ in scenarios that present increased risk of aerosol- and fomite-mediated disease transmission from feces^6–9^. The largest droplets settle out within seconds, but smaller aerosols ($<5\ \mu$m) remain suspended^10, 11^. The presence of pathogens on toilet bowl sidewalls or in bowl water contributes to contamination of the aerosols^4^, and contamination of bowl water may persist after dozens of flushes^12, 13^. Bioaerosol concentrations released from a flushed toilet vary depending on the type of toilet^14, 15^, ventilation performance^16^, radial position around the bowl^17^, water supply pressure level^18^ and the presence of fecal waste^11^. While growth of the aerosol plume is reduced—but not eliminated—by the presence of a closed lid^2, 10, 19^, toilets in public, commercial, or healthcare settings typically do not have lids. While previous studies document where toilet aerosols end up, very little is known about the physics and kinematics of how they get there.

While many epidemiological associations with fecal-oral sanitary contexts are well established, their aerosol counterparts are lacking. Respiratory exposures to airborne microbes in sanitary settings has been a public health focus as the availability of enclosed public toilets expanded with urbanization. Although quantitative risk assessments in this hygiene context have been constructed^20^, their practical application requires emission source characteristics as well as the time-resolved identity, distribution, abundance and specific persistence of potential pathogens aerosolized in respirable size ranges^21^. Sanitary aerosol exposures are acute, with a strong dependence on occupant behavior, which adds to the challenge of assessing respiratory risks in confined lavatory environments. The risks associated with transmitting respiratory and enteric viruses through use of public toilets due to contaminated aerosols from the toilet plume, suspended aerosol from prior users or transmission via high-touch surface should be mitigated where possible^4, 22, 23^. SARS-CoV-2 and other viruses have been shown to survive on surfaces for several days^24–26^, and enteric bacteria—potentially pathogenic and otherwise—such as *C. difficile* are aerosolized upon flushing and subsequently deposit onto local architectural surfaces as potential fomites^10^.

Current knowledge of toilet aerosol plumes derives mostly from discrete measurements of airborne and settled particle concentrations. Knowledge of the toilet plume kinematics is limited to high-speed video of large ejected droplets in close proximity to the bowl^8^, qualitative visualization of a dry ice plume from an aircraft lavatory^27^ and numerical simulations of flow velocities and expelled particles within an idealized toilet model^15^. Full-field, time-resolved measurements of the spatiotemporal plume dynamics—including the airflow velocity fields—are lacking, and are critical for development and testing of future design strategies to mitigate human exposure through plume formation, disinfect pathogens carried by the plume^28, 29^ and enhance plume removal through ventilation^20^.

## Results

**Figure 1:**
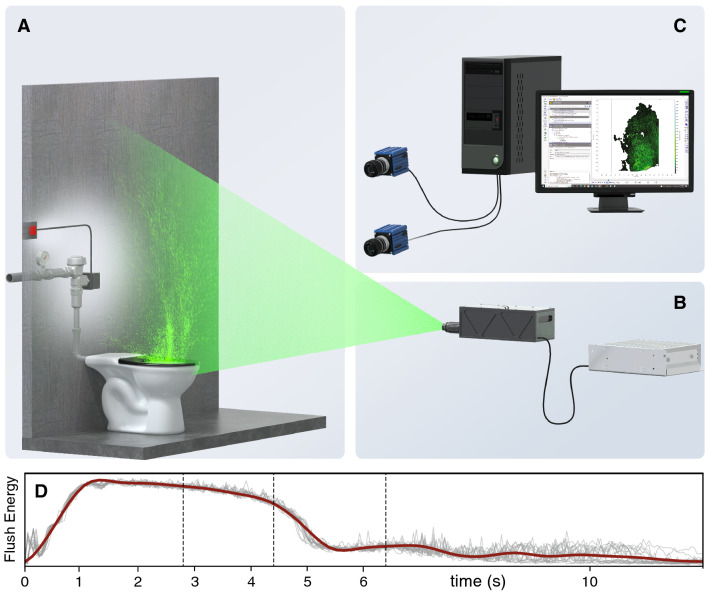
Laboratory illumination and imaging of flush-generated aerosol plumes. (**A**) Laboratory experiment with a flushometer-type (1.6 gallon per flush) commercial toilet. For all experiments, the toilet contains—and is flushed with—pure tap water with no solids or additives. The flush valve (located behind rear wall) is fed by a 60 psi supply and is electrically activated via a push button. (**B**) We use continuous and pulsed lasers to generate a thin light sheet that illuminates the plume in a vertical plane above the bowl centerline. (**C**) Laser light scattered by aerosol particles during and after the flush cycle are imaged by cameras. (**D**) Time history of flush cycle strength and duration using average sound pressure levels in the bowl (red line) from 20 flush replicates (gray lines) as a metric. The time *t*=0 corresponds to the instant when the flush button is pushed.

To visualize and quantify aerosol plume kinematics over a flushometer-type commercial toilet typical of those in use in North America (Fig. 1A), we use continuous and pulsed lasers to create a thin light sheet (Fig. 1B) that illuminates a vertical plane on the toilet centerline. When the toilet is flushed, the resulting aerosol plume scatters light that is imaged from the side with cameras (Fig. 1C). The toilet flush sequence begins with a remote button push that activates a solenoid valve on the flushometer, allowing supply water to enter the flushometer valve (in public restrooms this activation is often done by an optical motion sensor). Flush water entering the bowl creates an energetic turbulent flow with a familiar audible signature. We use sound pressure levels measured by a noise meter near the bowl as a metric for the strength and duration of the flush cycle (Fig. 1D). The flushing action lasts for approximately 5.5 seconds after the button is pushed ($t=$ 0), with most of the flush energy occurring for $0.5<t<5$ s; much of the sound recorded for $t>6$ s is associated with the bowl refilling after the flush.

**Figure 2:**
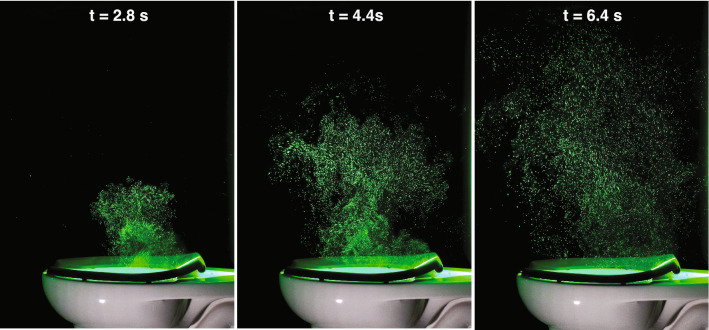
Photographs of the illuminated aerosol plume at $t=$ 2.8, 4.4, and 6.4 s (indicated with dotted lines in Fig. 1D) after flush initiation. For these images and for Movie S1, we used a continuous wave laser and a commercial color camera; the images show the illuminated plume as it appears to the human eye in the laboratory.

To demonstrate the structure of the illuminated aerosol plume in the laboratory as visible to the human eye, we used a continuous laser and a commercial digital camera to take a sequence of color images of the plume (Fig. 2). For these photos, we selected a slow shutter speed (1/50 s) to induce motion blur of particles in localized regions of high-velocity jet-like flow structures transporting the plume upwards from the bowl. The unsteady and dynamic nature of the plume is particularly evident in Movie S1.

To gather quantitative data about aerosol particle locations and motion, we use a pulsed laser and a pair of scientific sCMOS cameras to image light scattered by the particles. In this case the image exposure time (effective shutter speed) is set by the laser pulse duration (5 ns), resulting in sharp images with < 0.02 pixel of motion blur. Previous studies have shown that aerosol particle counts correlate with pathogen levels in the plume^18^, so the intensity of scattered light in our images is also expected to correlate with potential pathogen levels. For enhanced spatial resolution (250 $\mu$m), each camera captures separate—but slightly overlapping—regions of the plume; the two images are stitched together to form a single 0.57 m wide $\times$ 1.23 m high image that reaches 1.59 m above the floor.

**Figure 3:**
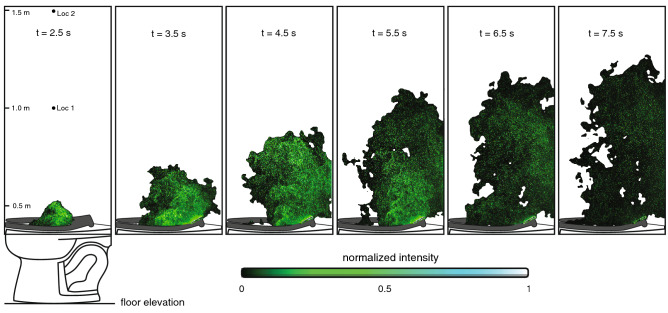
Spatial distribution and growth of aerosol plumes over time. Time sequence at 1 s intervals of flush-induced instantaneous aerosol plumes showing rapid and persistent growth. Each panel displays the intensity of imaged particles (green colormap) and computed plume envelopes (black regions), where the indicated times correspond to the flush sequence in Fig. 1D. The complex shape of the envelope boundary stems from the turbulent and chaotic flowfield induced by the toilet flush. The two locations “Loc 1” and “Loc 2” indicated in the first panel correspond to locations where optical particle measurements are taken, as shown in Fig. 5.

To map the spatial evolution of the flush-induced aerosol plume (Fig. 3), we use an intensity thresholding technique to compute plume envelopes at different times in the flush sequence, where the indicated times correspond to the flush cycle in Fig. 1D. The computed envelope (rendered in black) demarcates the boundary of the advancing front of aerosols. The intensity of imaged particles is rendered in green for visual consistency with the color photographs in Fig. 2. The plume is ejected upwards and rearwards towards the wall behind the toilet, with the highest concentration of particles occurring within jet-like structures in the interior region of the envelope. By *t* = 4.5 s, the plume impinges on the impermeable rear wall, impeding rearward motion and enhancing the vertical motion. The plume motion is chaotic and turbulent, as exhibited by the complex and fractal nature of the envelope, with frequent incursions of aerosol-free air intruding towards the plume interior. The imaged plume envelope rises to a height of over 1.3 m within 7.5 s. Repeated trials reveal envelope variations from flush to flush—the chaotic physics are sensitive to small differences in initial conditions—but the large-scale plume features are consistent. For times greater than 8 s, plume spreading and dilution with surrounding air limits our ability to directly image further growth of the plume. To demonstrate that the plume grows and persists for times > 8 s, we use a more sensitive airborne particle counting instrument, as discussed below.

**Figure 4:**
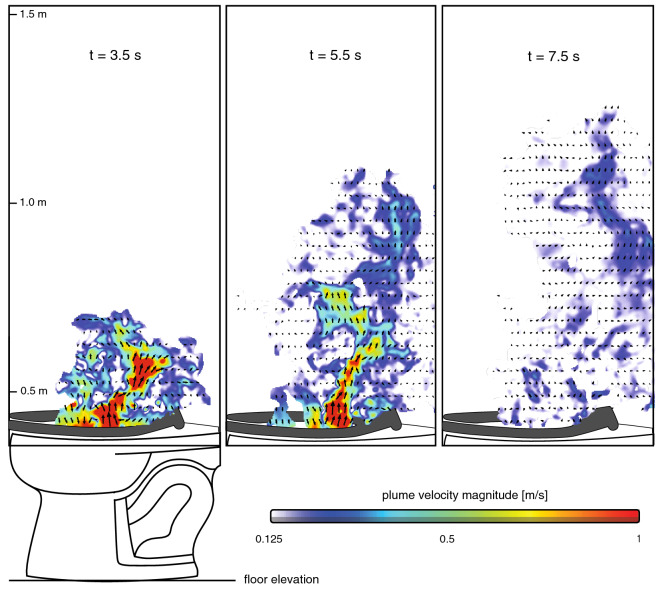
Turbulent flowfields responsible for aerosol plume transport. Time sequence of flush-induced instantaneous velocities computed from particle image pairs using PIV for the same flush sequence shown in Fig. 3. Flow direction is indicated within the plume envelopes by black arrows, and velocity magnitude is indicated by arrow length and also by the colormap. The turbulent velocity fields exhibit high velocity jet-like structures (red) that oscillate rapidly during the flush. The velocity magnitude colormap starts at 0.125 m/s which is typical of ambient flow velocities in the laboratory where the measurements were made.

To quantify the magnitude, direction, and structure of the flush-induced airflow that transports the aerosol plume, we use particle image velocimetry (PIV)^30, 31^ to compute instantaneous velocity fields within the plume envelopes (Fig. 4). Velocity direction and magnitude are represented by the orientation and length of the black arrows. For clarity, velocity magnitude is also indicated by the colormap overlay. The imaged aerosols serve as effective seeding particles for PIV, resulting in computed velocity fields with uncertainties generally < 5%. The flow is surprisingly energetic and chaotic, and exhibits strong jet-like behavior with velocities of 1 m/s or higher (red regions in the first two panels) that oscillate unpredictably during the first 6 seconds of the flush cycle. Instantaneous velocities greatly exceed the ambient flow velocities in the lab (10-15 cm/s) and occasionally exceeded 2 m/s. The large, nonuniform, and unsteady velocities efficiently transport and disperse aerosols ejected from the bowl during the flush.

**Figure 5:**
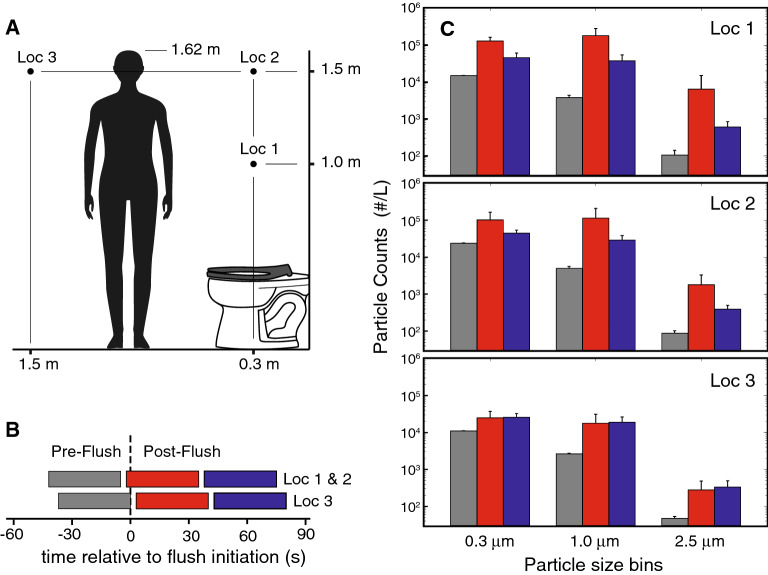
Size and number of ejected aerosol particles. (**A**) An airborne particle counter is used to take particle measurements at indicated locations; Locations 1 and 2 are also shown for reference in Fig. 3. The human figure provided for scale is 1.62 m tall, corresponding to the average height of an American female adult. (**B**) Particle counts are averaged over the indicated 37 s intervals, one pre-flush (gray bars), and two post-flush (red and blue bars). (**C**) Particle counter results are binned into three size categories for each of the three locations. The histogram bars represent the average of five replicates, and the listed size bins correspond to $0.2\ \mu \text {m}\le d\le 0.3\ \mu$m, $0.3\ \mu \text {m} < d \le 1.0\ \mu$m, and $1.0\ \mu \text {m} < d \le 2.5\ \mu$m.

To understand the potential for toilet plumes to expose humans to pathogens in aerosols, we use an optical particle counter to measure the size and quantity of aerosol particles ejected by the flush cycle^10, 11, 17^. Measurements are made at three locations (Fig. 5A): Locations 1 and 2 are over the bowl—and are also shown for reference in Fig. 3—while location 3 is further away from the rear wall. To see the effect of the flush cycle on particles at each location, we measured particle counts over three time intervals (Fig. 5B): one pre-flush interval (gray bars), one that starts immediately post-push (red bars) and one that is centered at 60 s post-flush (blue bars). Particle counts were grouped into three size bins: $0.2\ \mu \text {m}\le d\le 0.3\ \mu$m, $0.3\ \mu \text {m} < d \le 1.0\ \mu$m, and $1.0\ \mu \text {m} < d \le 2.5\ \mu$m; most particles were in the two smaller bins. Over the bowl (Loc 1 and Loc 2), particle counts increased by roughly an order of magnitude or more within 30 s of flush initiation, with counts decreasing afterwards. Further away (Loc 3), the particle count increase is more modest but remains constant through both of the post-flush time intervals.

## Discussion

Our results demonstrate the surprisingly energetic and rapid growth of aerosol plumes from a commercial toilet and highlight the chaotic nature of the fluid kinematics that transport the particles. The rapid spread of the plume is facilitated by a strong unsteady jet that angles upwards and backwards towards the rear wall; this jet is visible in the first frame of Figs. 2 and 4, and particularly so in Movie S1. Future toilet designs that reduce the strength of this jet—or alter its upward trajectory—might then reduce the amount of ejected aerosols.

For our experiments, the bowl contained only tap water with no solids present. The presence of fecal matter and toilet paper could alter plume dynamics in unpredictable ways. The experiments are performed in the center of a 300 $\text {m}^3$ ventilated laboratory space with no partitions, so measured aerosols are likely dispersed and diluted more rapidly than would be observed in a typical public restroom or toilet stall.

While traditional airborne particle counter measurements like those shown in Fig. 5 are effective at quantifying the size and quantity of ejected particles—and are therefore critical for quantifying potential pathogen exposure—they provide little understanding of how particles leave the bowl, what paths they follow, and how they get to the particle-counting locations. The methodologies we use to visualize and quantify the plume structure could provide a foundation for mitigating the risk of pathogen spread, by facilitating comparative studies of novel flush valve cycles and bowl geometries, and for quantifying particle paths and flight times in the context of human exposure and testing of disinfection strategies.

## Methods

### Toilet Rig

To measure toilet aerosol plumes in our laboratory, we use a commercial toilet with a common 1.6 gallon-per-flush flushometer-style valve, typical of those found across North America in public restrooms. The toilet is fitted with a commercial seat in the “down” position that is painted flat black to minimize laser reflections. There is no lid fitted to the toilet, consistent with most commercial toilet installations. The rear of the toilet abuts a solid wall; the flushometer valve and associated plumbing are behind this wall.

An electric pump fills a 14 gal water tank with an internal pre-charged air bladder system; the pump is set to shut off when the tank pressure reaches 60 psi, at which point the toilet is ready to be flushed (the flushometer valve has a recommended supply pressure between 10 and 100 psi). The tank is plumbed to the flushometer inlet, and the flush cycle is initiated via a remote button that activates a solenoid on the flushometer valve. During the flush, pressure in the tank drops ‘such that the supply pressure at *t* = 7.5 s (final panels in Figs. 3 and 4) has dropped to 45 psi. Following the flush cycle and data acquisition, the pump is activated to reset the tank pressure to 60 psi. The experiments are performed in an open laboratory space, and we rely on the laboratory HVAC system to ventilate flush-generated particles between experiments.

### Imaging with continuous laser

**Figure 6:**
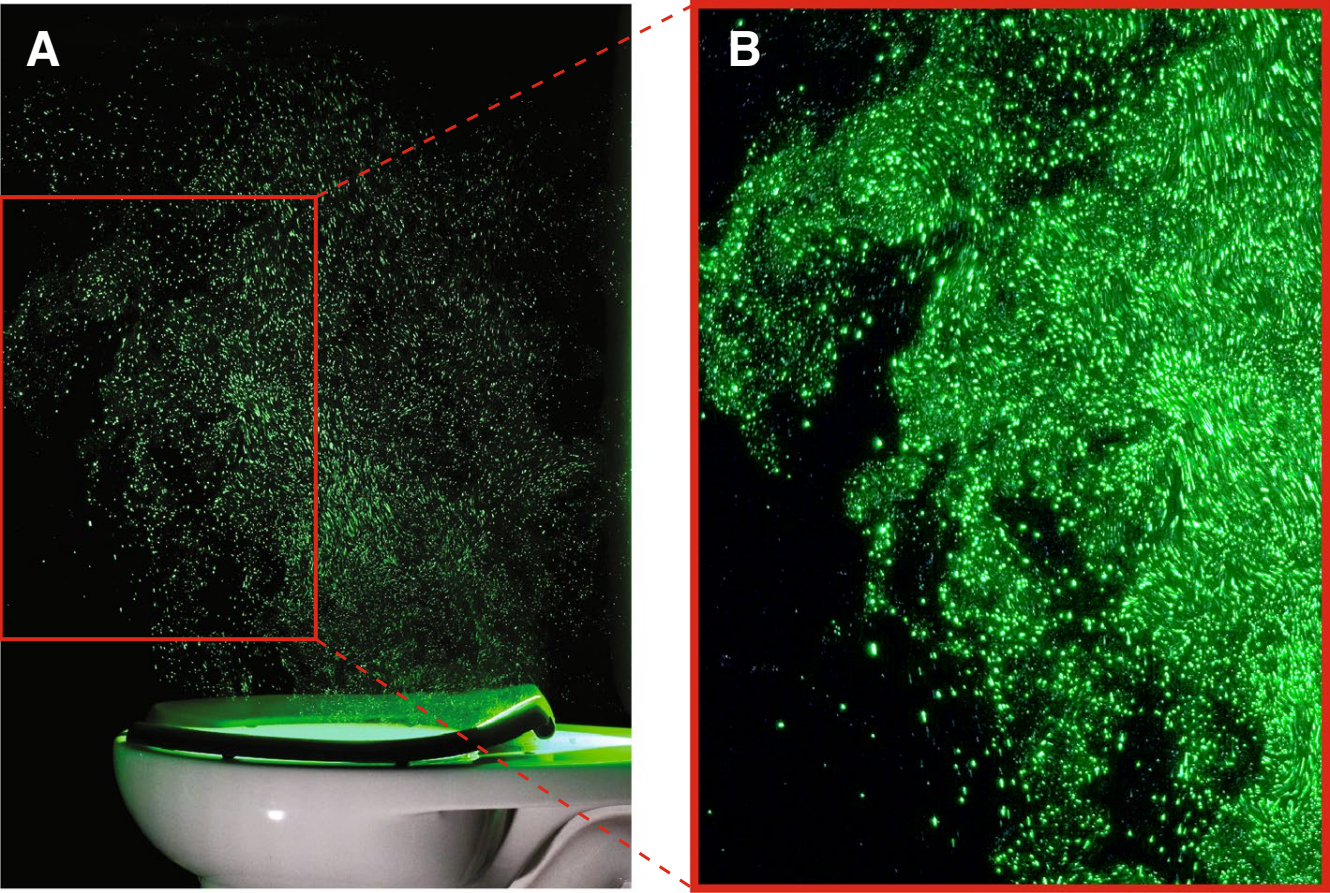
Imaged particles are good indicators of the plume envelope. (**A**) The third panel of the plume (*t* = 6.4 s) from Fig. 2 is reproduced here for reference, showing the location and motion of larger particles. (**B**) Enlarged image of the region denoted by the red box in part A, post-processed to increase exposure and decrease contrast. This renders dimmer light from smaller particles visible, and demonstrates that the smaller particles (green glow) are well predicted by the locations of larger particles (imaged as discrete points of light). Also visible in the lower left are several large droplets that are following ballistic trajectories and are outside the aerosol plume envelope.

To capture color images (Figs. 2 and 6) and video (Movie S1) of the aerosol plume, we use a continuous wave (CW) laser (IPG Photonics GLR-50, 532 nm wavelength, operating at a power level of 11 W) to illuminate a vertical plane aligned with the toilet’s axis of symmetry. Light sheet optics spread the beam into a sheet with a 2 mm beam waist centered on the field of view (FOV). Mie scattering of laser light by aerosol particles is imaged with a Sony camera (a6300) fitted with a Sony 50 mm f/1.8 lens. Still images are acquired with a 1/50 s shutter speed at 4000 x 6000 pixel resolution, and videos are acquired at 60 fps with 1/60 s shutter speed and 1920 $\times$ 1080 pixel resolution.

While our imaging technique is most effective at imaging the location and motion of larger aerosols (5-10 $\mu$m) that scatter more light, post-processing the images to increase brightness and reduce contrast (Fig. 6) demonstrates that it is also able to capture dimmer light scattered by smaller aerosols, and that the smaller particles move within the same envelope as the larger ones.

### Imaging with pulsed laser

To quantify the spatiotemporal evolution and kinematics of flush-induced aerosol plumes (Figs. 3, 4) we use a dual-cavity, double-pulsed Nd:YAG laser (Quantel EverGreen 200, 532 nm wavelength, operating at 200 mJ/pulse). The laser emits pairs of pulses ($dt=$ 2.25 ms), each with a 5 ns pulse width. Pulse pairs are repeated at 15 Hz. As with the CW laser, we use sheet optics to create a 2 mm light sheet spanning the FOV. Images are acquired using two sCMOS cameras (LaVision Imager sCMOS; 16-bit monochrome, 2160 $\times$ 2560 pixel resolution) fitted with Nikkor 50 mm f/1.2 lenses. The cameras are stacked vertically (Fig. 1C), with the long axis of the sensors oriented vertically and pointed such that there is a 15% overlap in the FOV of each camera; this permits individual images to be stitched together to provide a combined FOV (0.57 m wide $\times$ 1.23 m high) that is large enough to capture the entire toilet plume during the first 8 s following flush initiation, with a spatial resolution of 260 $\mu$m/pixel. The narrow depth-of-field associated with the f/1.2 lens aperture, along with the thin light sheet and 5 ns illumination pulses permits selective imaging of scattered light from in-sheet aerosols for subsequent computation of plume envelopes and aerosol velocities.

A large spatial calibration target consisting of a high-contrast square grid covering the entire FOV of the combined camera set is used (i) to compute the optical magnification of the imaging system, (ii) to reference the image data to the toilet geometry in physical space, and (iii) to map each camera to a common point in physical space, allowing for the accurate stitching of the two individual FOVs into a single data image covering the total resolved FOV. The known geometry of the grid on the calibration plate is also used to create a pinhole model^32^ to dewarp the individual camera images, correcting for potential image distortions associated with small oblique imaging angles and/or lens distortions. The pinhole model is appropriate for our planar imaging configuration with undisturbed optical access through air. The estimated uncertainty in the reconstructed/combined image data is less than 0.5 px.

Closely-spaced (2.25 ms separation) pairs of images are acquired from each camera at a rate of 15 Hz during flush events. The images are used to quantify the spatiotemporal evolution of the plume envelope (Fig. 3) and to compute aerosol velocities (Fig. 4) using particle image velocimetry^30, 31, 33^. All laser and camera timing sequences and associated image acquisition, storage, and processing are achieved on a high-performance computer running DaVis 10.2 software (LaVision GmbH).

### Imaging system resolution

For the 260 $\mu$m/pixel resolution of our optical system (described above), individual aerosol particles (0.1 $\mu$m - 10 $\mu$m) are only a small fraction of the imaged size of an individual pixel. However, for our low-magnification optical system, aperture diffraction^34, 35^ causes the imaged size of these aerosol particles to increase to at least a theoretical minimum of approximately 0.25 pixel, regardless of their physical size. Then, lens aberrations further enlarge the theoretical minimum diffraction size by as much as an order of magnitude, particularly for systems with large working distances (> 1 m) as is the case for ours ($\approx$ 2 m). Digitization and quantization of the continuous particle image signal onto a discrete pixel grid can also enlarge the recorded particle size. Thus, individual aerosol particles are expected to produce imaged spots that are several pixels or more in diameter. Furthermore, given that the true size of individual aerosol particles is tiny compared to the pixel resolution of our system, it is reasonable to expect that the light gathered by a single pixel is due to a large number of particles, all of which contribute to the imaged intensity at that point. Consistent with these arguments, our recorded images exhibit strong particle images with typical diameters $d_D$ of 1.5 to 4 pixels (see PIV section below). The result is that the strong multi-pixel images of particles (or even large numbers of particles) are well suited for instantaneous mapping of the spatial envelope of the aerosol plume (Fig. 3) and computing aerosol velocities (Fig. 4). However, the same optical properties that render the optical system suitable for these tasks preclude its use for counting and sizing of individual aerosols. For this reason, counting and sizing are done separately with the airborne particle counter (Fig. 5).

### Generation of plume envelopes

The spatial extent of the aerosol plume envelope is computed from image data using a simple two-step image processing algorithm commonly used in PIV applications to delineate seeded and unseeded regions. First, a sliding maximum filter is applied to fill in regions of low pixel intensity between individual particle images. With appropriate filter length selection, the effect is to both increase and homogenize pixel intensities inside the plume envelope with minimal effect on regions outside the plume. Then a global intensity threshold is used to identify the in-plume versus out-of-plume regions (since higher pixel intensities correspond most notably to the presence of plume aerosols, as well as the size and local density of aerosols). While large changes in the tuning parameters produce local artifacts (e.g., unwarranted internal voids, or excessive perimeter smoothing), the general shape and spatial extent of the plume is generally robust for a range of tuning parameters.

### Determination of velocity fields with PIV

Particle image velocimetry (PIV) is used to compute aerosol velocities inside the detected plume envelope^33^. Here, each sCMOS camera acquires double-frame image data, where pairs of images are acquired at the imaging frequency of 15 Hz, which sets the temporal resolution of the velocity measurements. The image pair itself is separated by a short time *dt*, the cross-correlation timescale of the PIV analysis. A near-optimal *dt* results in maximum particle image displacements of approximately 8–10 px for a 32 px correlation subwindow ( the “1/4-rule” of^37^) based on the optical magnification of the imaging system and the physical velocities associated with particles. Here, a *dt* of 2.25 ms (set by the time delay between laser pulse pairs) is effective at resolving the high velocities (1 - 2 m/s) associated with the strong vertical jet that develops early in the flush cycle (Fig. 3) and minimizing the associated velocity uncertainty.

Particle signal level relative to background (intensity counts), particle seeding density (particles per pixel), and particle image diameter (pixels) strongly influence the resolved dynamic velocity range (DVR)^36^. Our imaging configuration yields signal levels (post background subtraction) of 8–10 bits ensuring good particle fidelity and strong intensity correlations. Seeding densities are set by the spatiotemporally evolving local density of aerosol clouds (and limitations of the imaging system resolution described above) and typically range between 0.001 and 0.02 ppp within the plume envelope. These densities span the commonly-accepted value of 0.01 ppp, sufficient to provide strong cross-correlation peaks and acceptable uncertainty levels. Finally, typical particle image diameters of $d_D=$ 1.5 to 4 px are sufficient to alleviate “peak locking” effects (integer pixel displacements) when implementing window-shifting correlation techniques as described below^38^. Image sets are first pre-processed to remove background artifacts and to enhance particle fidelity. Aerosol displacement (velocity) fields are then computed using modern digital correlation and interrogation techniques described below. Image preprocessing, PIV correlation analyses, and vector postprocessing are achieved on a high-performance computer running DaVis 10.2 software (LaVision GmbH).

Aerosol velocity fields (Fig. 4) are computed from image pairs via cross-correlation of intensity patterns within small interrogation subwindows inside the detected plume envelopes. Best practices are used to maximize the measurement DVR including multi-pass iterative schemes with overlapping (50% - 70%) subwindows of decreasing sizes (96 px - 32 px), adaptively shaped to local flow conditions. The resulting vector fields are then post-processed using an imposed minimum correlation peak ratio (1.4) to detect outliers and noisy vectors, which are discarded. The peak ratio is the ratio of the strongest correlation peak to the next strongest in a given interrogation window, and correlates well with the estimated measurement error^39^, providing a proxy for the effective DVR of the measurement. Finally, any gaps in the post-processed vector fields are filled using spatial interpolation and nonlinearly smoothed to preserve local gradients. The resulting vector field is space-filling inside the plume envelope with a spatial resolution of 2.08 mm/vector. Typical maximum local velocity uncertainties are less than 5% of the local velocity magnitude and rarely approach 10% (estimated using correlation statistics^40^). Corresponding peak ratios typically exceeding 10 throughout the plume envelope confirm the high DVR and good fidelity of the aerosol velocity measurements.

In traditional PIV applications, low Stokes number particles are introduced into the flow as passive tracers (“seeding particles”) whereas here we use the aerosols themselves to serve as natural tracers. The Stokes numbers (*St* = ratio of particle inertial timescales to the advection timescales of the flow) of 1-micron particles in the highest-velocity portions of the plume (around 1 m/s) are O(1). While larger aerosols around 10-micron exhibit higher *St* in these regions, the predominantly vertical velocities associated with the strong jet suggest that aerosol clouds of the sizes of interest here (0.1 - 10 microns) behave acceptably as passive tracers. The implication of all of the above for the PIV-based velocity is as follows: individual velocity vectors represent the mean velocity averaged over small clouds of aerosols (producing individual particle images on the sensor) and over collections of discrete aerosol clouds (producing all the particle images contained in a PIV interrogation subwindow).

### Sound measurements

As a metric for flush duration and intensity we acquire sound pressure levels with dB(A) frequency weighting using a Google Pixel 3 smartphone with a pre-calibrated mobile application (Decibel X, SkyPaw Co., Ltd.). Twenty replicates of a 12 second interval surrounding the flush cycle are recorded, where $t=0$ corresponds to when the flush button is pushed. The average sound pressure profile shown in Fig. 1D is smoothed with a cubic B-spline, downsampling by a factor of 10 to capture the characteristic shape. A second spline was applied with the original number of samples for smoothing.

### Airborne particle counting

Particle counting is done with a portable airborne particle counter (Particle Measuring Systems HandiLaz Mini II) suspended in the locations shown in Fig. 5A. The counter is sensitive to particles ranging in size from $0.2\ \mu$m (d50) to $10\ \mu$m, and outputs counts in 60 discrete logarithmically-spaced bins across this range. For clarity, we grouped particle counts from the instrument into the three broader bins shown in Fig. 5; particle sizes larger than $2.5\ \mu$m are not shown since these counts were close to zero. The counter ingests 2.83 liters/min, and the nozzle is oriented downwards, since the plume was generally approaching from below. For each location, particles are counted over three 37 s intervals (Fig. 5B). Each interval consists of five 5s samples separated by 3 s periods when the data is written to storage. The timing of the intervals at location 3 differs slightly from those in locations 1 and 2 due to phase differences in the discrete sampling by the instrument. Five replicates are acquired, with average and arithmetic standard deviations reported in Fig. 5C.

## Supplementary Information

Supplementary Information 1.

Supplementary Information 2.

## Data Availability

The datasets generated during and/or analyzed during the current study are available from the corresponding author on reasonable request.
