# Supplementary material for: Commercial toilets emit energetic and rapidly spreading aerosol plumes
Source: Sci Rep. 2022 Dec 8;12:20493. doi: 10.1038/s41598-022-24686-5 (PMC9732293; doi:10.1038/s41598-022-24686-5)
Supplement: Supplementary file 1 — Supplementary Information 1. [file 41598_2022_24686_MOESM1_ESM.pdf]

Movie S1: Laser illumination of aerosol plume ejected from a commercial toilet. Color real-time movie of the first 8 s following flush initiation, analogous to the still images in Fig. 3. The movie illustrates the energetic flapping of the chaotic jet emanating from the bowl, as well as the rapid spread of the aerosol plume.
